# The Transition From Maternity Blues to Full-Blown Perinatal Depression: Results From a Longitudinal Study

**DOI:** 10.3389/fpsyt.2021.703180

**Published:** 2021-11-03

**Authors:** Mario Luciano, Gaia Sampogna, Valeria Del Vecchio, Vincenzo Giallonardo, Francesco Perris, Marco Carfagno, Maria Luce Raia, Matteo Di Vincenzo, Marco La Verde, Marco Torella, Andrea Fiorillo

**Affiliations:** ^1^Department of Psychiatry, University of Campania, “L. Vanvitelli”, Naples, Italy; ^2^Department of Woman, Child and General and Specialized Surgery, Obstetrics and Gynaecology Unit, University of Campania “Luigi Vanvitelli”, Naples, Italy

**Keywords:** maternity blues, perinatal depression, EPDS, anxiety symptoms, risk factor

## Abstract

**Background:** The aims of the present study are to: (1) assess the frequency of maternity blues (MB); (2) identify the clinical and social characteristics more frequently associated with the onset of depressive symptoms after delivery; and (3) verify the hypothesis that the presence of maternity blues is a risk factor for the onset of a full-blown depressive episode in the 12 months after delivery.

**Methods:** This is a longitudinal observational study. All pregnant women who gave birth at the inpatient unit of Gynecology and Obstetrics of the University of Campania “Luigi Vanvitelli” from December 2019 to February 2021 have been invited to participate in the study. Upon acceptance, women were asked to complete the Italian version of the Edinburgh Postnatal Depression Scale along with an *ad-hoc* questionnaire on the women's sociodemographic, gynecological and peripartum characteristics as well as their psychiatric history. Women have been reassessed after one, 3, 6 and 12 months.

**Results:** A total of 359 women were recruited within 3 days from delivery, with a mean EPDS total score of 5.51 (±4.20). Eighty-three women (23.1%) reported the presence of maternity blues. Mean EPDS total scores were 12.8 (±0.2) in the MB group vs. 4.26 (±0.2) in the group without MB (*p* <0.0001). MB predictors were the presence of an anxiety disorder with an onset 6 months prior to pregnancy, of preeclampsia, of increased fetal health rate, of conflicts with relatives other than partner and having a partner with an anxiety disorder. At multivariate analyses the presence of MB increased 7-time the risk to have a higher EPDS score at follow-up assessments (OR: 7.79; CI: 6.88–8.70, *p* <0.000). This risk is almost four times higher 1 months after the delivery (OR: 4.66; CI: 2.54–6.75, *p* < 0.000), almost three times higher after 3 months (OR: 2.98; CI: 0.50–5.46, *p* < 0.01) and almost six times higher after 12 months (OR: 5.88; CI: 3.20–8.54, *p* < 0.000).

**Conclusions:** Although MB was a self-limiting condition in the majority of cases, depressive symptoms arose quite often immediately after the childbirth. Professionals should be trained to monitor symptoms of MB and its transition toward a depressive episode.

## Introduction

Depressive and anxiety disorders are the most frequent mental disorders occurring in the postpartum period and are associated with a high personal burden for the mother and the newborn (1–3). Moreover, the onset of depressive and anxiety symptoms is associated with subsequent childhood morbidity in 75% of mothers who have depressive and/or anxiety symptoms with an onset in the perinatal period (4).

Recent systematic reviews and meta-analyses reported that the onset of a clinically relevant depressive episode occurs in 12% of mothers within the postpartum period, with a higher prevalence in low- and middle-income countries (5, 6). The rates of depressive and anxiety symptoms with an onset immediately after delivery are significantly higher than in the subsequent period of post-partum. These symptoms are also referred to as “maternity blues” or “baby blues,” characterized by depressive symptoms, insomnia, anxiety symptoms, and tearfulness. Maternity blues (MB) is not considered a mental disorder *per se*, but it is conceptualized as an episodic psychological distress, usually self-limiting without any treatment. A widely accepted definition of maternity blues is not yet established, and a diagnostic differentiation with a depressive episode with onset in the postpartum period is still needed (7, 8). In fact, if from one hand MB is considered a mood disorder with overlapping clinical characteristics with the classical postpartum depression, several authors suggested that MB is mainly characterized by anxiety symptoms and not exclusively by depressive symptoms (9). Other authors consider MB an “atypical” depression, characterized by a prevalence of neurotic symptoms, such as anxiety, irritability and phobias (10–12).

The prevalence rates of MB greatly vary among studies, ranging from 10 to 80% (13). A recent metanalysis of 26 papers on a total of 5.667 participants reported that the pooled prevalence rate of maternity blues is 39%, with higher prevalence rates in low- and middle- income countries (76 and 40.8%, respectively) (14). However, this high heterogeneity in the prevalence rates of MB largely depends on the lack of a clear diagnostic definition and on the absence of definite boundaries between MB and the normality (15). It should be noted that research has been influenced by the conceptualization of maternity blues as a self-limiting condition, and therefore only a few studies have showed the real impact of the presence of depressive and anxiety symptoms on the well-being of mothers and their children (16).

Despite maternity blues is not considered an illness entity, several findings have documented its possible consequences, including the high risk of developing a full-blown depressive disorder in the mothers during the postpartum period and mental health problems in children (17, 18). In fact, about 20% of women presenting symptoms of MB receive a diagnosis of postpartum depression (PPD) in the first year after delivery (19, 20). Moreover, in a sample of 1,024 mothers, Reck et al. (21) reported that the onset of maternity blues is associated with an increased risk of developing a DSM-IV postpartum depressive or anxiety syndrome. The predictive role of maternity blues is particularly high in primiparae that in mothers who have already other children (22).

Only a few studies have showed the negative effects of MB on child development. However, evidence shows that the presence of depressive symptoms with an onset in the first phases of the puerperium may affect the mother-child relationship, having a detrimental effect on the cognitive and emotional growth of the infant (21, 23, 24). Children of women with depression are at increased risk of having an insecure attachment, difficulties in interacting with others and delays in the competencies' acquisition (25–27). Moreover, frequently women with MB tend not to start or discontinue breastfeeding very early and have a significantly reduced interaction with the newborn (28, 29). All studies converge on the fact that, if untreated, MB symptoms can have serious consequences on women's and infants' mental health (30, 31).

In this paper we report the results of a longitudinal study whose main novelties are the identification of clear predictors of maternity blues in a significant sample of women and the assessment of the relationship between maternity blues and the risk to develop depressive symptoms up to 12 months after delivery. To our knowledge only few studies have been carried out on these topics.

Therefore, our aims are to: (1) assess the frequency of depressive symptoms immediately after delivery; (2) identify the clinical and social characteristics more frequently associated with the onset of depressive symptoms after delivery; and (3) verify the hypothesis that the presence of maternity blues is a risk factor for the onset of a depressive episode in the 12 months after delivery.

## Methods

This is a longitudinal observational study carried out at the Gynecology and Obstetrics unit in collaboration with the Department of Psychiatry of the University of Campania “Luigi Vanvitelli.” All pregnant women who gave birth in the period December 2019–February 2021 were invited to participate in the study. Women with a severe intellectual disability or with a pre-existing diagnosis of schizophrenia, schizoaffective disorder, delusional disorder, other not specified psychosis-spectrum disorder or with bipolar disorder were excluded.

Patients were recruited within 3 days after the delivery. After acceptance, women were asked to complete the Italian version of the Edinburgh Postnatal Depression Scale (EPDS) (32). At baseline, the following information were collected for all enrolled women: (1) sociodemographic characteristics (age, nationality, educational level, marital and employment status; previous pregnancies); (2) gynecological history (spontaneous or voluntary previous abortions, previous pregnancies, mental disorders during previous pregnancies); (3) clinical information about current pregnancy (vaginal vs. cesarean delivery, presence of obstetric complications during pregnancy including eclampsia, gestational diabetes, epidural analgesia, episiotomy, 1 and 5 min Apgar Score and oxytocin augmentation); (4) psychiatric history (presence of pre-existing mental disorders, drug/alcohol abuse, use of psychotropic drugs); (5) social and contextual factors (relationship with the partner, family conflicts, socio-economic situation, presence of life-time stressors).

The EPDS was compiled by women after 1, 3, 6, and 12 months after the delivery. The EPDS is the most frequently used instrument to assess depressive symptoms during pregnancy and in the post-partum period, although its use after 12 months from childbirth is less common. However, we decided to use the same instrument, rather than opting for an assessment instrument more frequently used in adult depression, in order to obtain comparable data in all study timepoints.

The Edinburgh Postnatal Depression Scale (EPDS) is a10-item self-reported screening questionnaire initially developed for use in the postnatal period to improve detection of postnatal depression. Each item is rated on a 4-point Likert scale (0–3). EPDS has satisfactory sensitivity and specificity values, and it is also sensitive to changes in the severity of depression over time. In validation studies, different cut-off points have been found. The cut-off score for severe depressive symptoms (i.e., a positive screening result) is ≥13 points but, as suggested by Hewitt et al. (33), a cut-off score ≥ 10 is usually adopted for screening purposes and can be used in order to identify patients with major or moderate depressive symptoms. Therefore, in this study we used a cut-off of 10 or above to identify women with maternity blues.

This study has been carried out in accordance with globally accepted standards of good clinical practice, in agreement with the Declaration of Helsinki and with national and local regulations. The study investigators ensured that all mental health professionals involved in the study are qualified and were informed about the protocol and trial-related duties. The study protocol was approved by The Ethical Review Board of the University of Campania “Luigi Vanvitelli” (protocol number 98 of February 28, 2019).

### Statistical Analyses

Descriptive statistics were performed for the socio-demographic and clinical characteristics of the sample, as well as to report the percentage of women presenting maternity blues or perinatal depression. According to the study protocol, the time points of data collection were recorded and coded using the variable “time.” The cut-off at EPDS ≥10 was adopted to divide the sample in two groups, those with and without MB. Moreover, according to Tuohy and McVey (34), three subscales were extracted: anhedonia (items 1 and 2), anxiety (items 3 to 6) and depression (items 7 to 10). In order to identify the clinical, sociodemographic and gynecological characteristics more frequently associated with maternity blues, chi-square analyses for categorical variables were performed; two-tailed *t*-test analyses for continuous variables were also performed to compare women with and without symptoms of maternity blues. A logistic regression model was carried out to identify the risk factors for MB. All variables that were statistically significant at the univariate analyses were entered as independent variables in the multivariate analyses.

Furthermore, in order to test the hypothesis that the presence of maternity blues can be considered a reliable predictor of EPDS mean scores at follow-ups, a linear regression model was performed, with the EPDS total score as dependent variable. The presence of maternity blues was entered in the model as independent variable as well as the categorical variable “time” (including all follow-up assessments). The model was adjusted for several socio-demographic, clinical and contextual characteristics, as well as for obstetric and neonatal outcomes more frequently associated with maternity blues and/or perinatal depression. Statistical analyses were performed using the Statistical Package for Social Sciences (SPSS), version 17.0. For all analyses, the level of statistical significance was set at *p* < 0.05.

## Results

A total of 359 (out of 412 contacted; participation rate=87%) out women were enrolled within 3 days from the delivery. Main reasons for refusal to take part in the study were: (1) not being interested in participating in the study (*N* = 31; 58.5%); (2) worries about feeling stigmatized (*N* = 15, 28.3%); (3) discharge from the hospital after 1 day from childbirth (*N* = 7; 13.2%). The participation rate of 87% is acceptable and comparable with those found in other studies (21, 35, 36). No statistically significant differences were reported among women who refused to participate and those enrolled in the study in terms of socio-demographic characteristics. Enrolled women had 32.1 (±6.0) years and a good level of education (40.3% had a secondary school degree and 33% a university degree). Half of them were employed, 5.6% had a positive psychiatric history of depressive disorders and 8.5% of anxiety disorders, requiring any psychopharmacological or psychological treatment. 28.7% of women had a previous abortion, 7% reported conflicts with the partner and 6.8% with other family members. In our sample, 4.3% (*N* = 15) of women reported preeclampsia during the current pregnancy and 2% (*N* = 7) an increase in fetal heart rate. The mean duration of labor was 7.34 h (±9.42; intended as admission at 3 to 4 cm dilatation in the presence of uterine contractions with or without rupture of membranes); the 5 min Apgar was 9.3 (±0.9). Women reported a mean EPDS total score of 5.5 (±4.2), a mean EPDS anhedonia subscale score of 0.7 (±1.0), a mean EPDS anxiety subscale of 2.7 (±2.1) and a mean EPDS depression subscale of 1.4 (±1.8). All socio-demographic, clinical and obstetric variables of the sample are reported in Table 1.

**Table 1:** Socio-demographic and clinical characteristics of the sample.

	**Total sample (*N* = 359)**	**EPDS > 10 (*N* = 83)**	**EPDS <10 (*N* = 276)**
**Level of education, % (** ***N*** **)**			
Primary School	2.5 (9)	1.9 (2)	2.5 (7)
Secondary School	24.2 (86)	30.2 (25)	22.9 (61)
High School	40.3 (147)	39.6 (33)	41.3 (114)
Degree	33.0 (117)	28.3 (25)	33.3 (92)
Employed, yes % (*N*)	50.6 (178)	43.4 (36)	51.4 (142)
History of depressive disorders prior to pregnancy, yes, % (*N*)	5.6 (20)	18.9 (16)	1.4 (4)^****^
History of anxiety disorders priori to pregnancy, yes % (*N*)	8.5% (30)	30.2 (25)	1.8 (5)^****^
Tobacco use, yes 5 (*N*)	7.0% (25)	9.4 (8)	6.1 (17)
Previous abortions, yes, 5 (*N*)	28.7% (100)	37.7 (31)	25 (69)
Conflicts with parents, yes, % (*N*)	6.8 (24)	19.2 (16)	2.8 (8)^***^
Conflicts with partner, yes, % (*N*)	7.0 (25)	11.5 (10)	5.4 (15)^*^
Financial problems, yes, % (*N*)	5.4 (19)	11.5 (10)	3.2 (9)^*^
Being victim of an assault 6 months prior to pregnancy, yes, % (*N*)	3.9 (17)	13.5 (11)	2.5 (7)^***^
Having a partner with depression, yes % (*N*)	2.6% (9)	4 (3)	2.2 (6)
Having a partner with an anxiety disorder, yes % (*N*)	6.3 (22)	16 (13)	3.2 (9)^**^
Vaginal delivery, yes, % (*N*)	67.3 (234)	57.7 (48)	67.4 (186)
Preeclampsia, yes, % (*N*)	4.3 (15)	15.4 (13)	0.72 (2)^****^
Increased fetal heart rate, yes, % (*N*)	2.0 (7)	7.8 (6)	0.36 (1)^*^
Need for neonatal intensive care, yes, % (*N*)	6.4 (22)	9.6 (8)	6.1 (17)
Age, M (sd)	32.12 (6.0)	31.26 (5.97)	32.26 (5.98)
BMI prior to pregnancy, M (sd)	24.86 (5.9)	24.19 (4.92)	24.98 (4.76)
Duration of labor, hours, M (sd)	7.34 (9.42)	6.25 (7.29)	7.53 (9.74)
Apgar index, 1 min, M (sd)	8.08 (1.4)	7.64 (1.79)	8.16 (1.41)^*^
Apgar index, 5 min, M (sd)	9.30 (0.85)	9.04 (0.95)	9.35 (0.86)^*^
Edinburgh Postnatal Depression Scale, total score, M (sd)	5.51 (4.20)	12.80 (0.16)	4.26 (0.16)^****^
Edinburgh Postnatal Depression Scale, anhedonia subscale, M (sd)	0.70 (1.0)	1.65 (1.44)	0.55 (0.81)^****^
Edinburgh Postnatal Depression Scale, anxiety subscale, M (sd)	2.69 (2.09)	5.67 (1.44)	2.15 (1.72)^****^
Edinburgh Postnatal Depression Scale, depression subscale, M (sd)	1.36 (1.79)	3.78 (2.27)	0.95 (1.26)^****^

*EPDS, Edinburgh Postnatal Depression Scale; BMI, Body Mass Index*.

* *p < 0.05;*

** *p < 0.01;*

*** *p < 0.001;*

**** *p < 0.000*.

### Differences Among Women With and Without MB

Eighty-three women (23.1%) reported the presence of maternity blues. In the MB group the mean EPDS total score was 12.8 (±0.2) vs. 4.3 (±0.2) in the group without MB (*p* < 0.0001). All EPDS subscales were significantly higher in the MB group (anhedonia: 1.6±1.4 in the MB group vs. 0.5 ± 0.8 in women without MB, *p* < 0.0001; anxiety: 5.7 ± 1.4 vs. 2.1 ± 1.7, *p* < 0.0001; depression: 3.8 ± 2.3 vs. 0.6 ± 1.3, *p* < 0.0001).

Compared to those without MB, women reporting depressive symptoms immediately after the delivery had more frequently a positive history of depressive disorders (18.9% in the MB group vs. 1.4% in women without MB, *p* < 0.0001) and of anxiety disorders prior to pregnancy (30.2% vs. 1.8%, *p* < 0.0001). Moreover, women with MB reported more frequently the occurrence of preeclampsia during pregnancy (15.4% vs. 0.7%, *p* < 0.0001) and the increase of fetal heart rate (6% vs. 1%, *p* < 0.05). The presence of conflicts with the partner and with other family members is more frequent in the MB group (11.5% vs. 5.4%, *p* < 0.05 and 19.2% vs. 2.8%, *p* < 0.001, respectively). Furthermore, women with MB reported more frequently to have a partner suffering from an anxiety disorder (16% vs. 3.2%, *p* < 0.01) and with financial problems (11.5% vs. 3.2%, *p* <05).

In the logistic regression model (Table 2), the likelihood to have MB was increased by the presence of: (1) history of anxiety disorder needing a psychiatric treatment (OR: 3.16; CI: 0.60–10.41, *p* < 0.0001); (2) preeclampsia (OR: 6.64; CI: 1.75–25.17, *p* < 0.01); (3) increased fetal health rate (OR 9.15; CI: 1.66–50.32, *p* < 0.01); (4) conflicts with relatives other than partner (OR 6.06; CI: 2.08–17.66, *p* < 0.001); (5) having a partner with an anxiety disorder (OR 4.39; CI: 1.37–14.09, *p* < 0.01).

**Table 2:** Predictors of Maternity Blues.

			**95% Confidence interval**	
	**OR**	***P*-value**	**Lower bound**	**Upper bound**
Constant	57.41	0.078		
Age	0.957	0.152	0.90	1.02
**Level of education, ref category: primary school degree**				
Secondary school degree	2.33	0.378	0.36	15.25
High school degree	1.94	0.49	0.30	12.63
University degree	1.23	0.83	0.17	8.71
History of depressive disorder prior of pregnancy	0.39	0.146	0.11	1.39
History of anxiety disorders prior to pregnancy	3.16	0.000	0.60	1.41
Having a partner with an anxiety disorder	4.39	0.010	1.37	14.09
Conflicts with relatives other than partners	6.06	0.001	2.08	17.66
Conflicts with partner	0.83	0.781	0.23	3.00
Financial problems	1.66	0.465	0.43	6.26
Being victim of an assault during pregnancy	1.36	0.815	0.11	17.42
Preeclampsia	6.64	0.010	1.75	25.17
Increased fetal heart rate	9.15	0.010	1.66	5.32
Apgar after 1 min	1.07	0.723	0.74	1.56
Apgar after 5 min	0.72	0.305	0.38	1.35

*OR, Odd Ratio*.

### Longitudinal Assessments

Of the 359 women assessed within 3 days after delivery, 178 returned the questionnaire after 1 month (T1), 161 after 3 months (T2), 109 after 6 months (T3), and 106 after 12 months (T4). The overall retention rate at 12 months follow up was 30%. In the majority of cases women did not complete the follow-up assessments since they did not answer to three consecutive phone calls (*N* = 140; 55.3%). Other reasons included lack of time (*N* = 70; 27.7%) and lack of interest (*N* = 20; *N* = 7.9%). Moreover, 23 women (9.1%) did not provide any reason. Globally, our retention rate is comparable with those reported by other studies adopting a similar methodology (22, 37).

No statistically significant differences were found among women who completed the 12 months follow-up assessments and those who did not in terms of age (31.7 ± 5.9 years in those who completed the EPDS after 12 months vs. 32.5 ± 6.02 years in drop-outs, *p* = 0.202), BMI prior to pregnancy (25.2 ± 4.6 vs. 24.6 ± 5.0, *p* = 0.239), duration of labor (7.7 ± 9.9 vs. 7.0 ± 9.0 h, *p* = 0.487), EPDS total score immediately after the childbirth (5.9 ± 4.4 vs. 5.2 ± 3.9, *p* = 0.113), positive psychiatric history of depressive disorders (8.6% vs. 7.9%, *p* = 0.210) and of anxiety disorders prior to pregnancy (9.1% vs. 7.6%, *p* = 0.356), presence of financial problems (7.6% vs. 3.3%, *p* = 0.099), conflicts with partner (9.3% vs. 4.9%, *p* = 0.089), and with other family members (7% vs. 6.6%, *p* = 0.521), presence of anxiety disorders in the partner (4.8% vs. 7.7%, *p* = 0.179), type of delivery (vaginal, 64.6% vs. 65.2%, *p* = 0.526), and presence of complications during pregnancy (yes, 23.7% vs. 27.7%, *p* = 0.219).

According to EPDS total score, the prevalence of post-partum depression was 17.4% (*N* = 31) at T1, 19.2 % (*N* = 31) at T2, 16.5% (*N* = 18) at T3 and 17.9% (*N* = 19) at T4. The linear regression model showed that the presence of maternity blues is a risk factor for high EPDS scores at follow-ups (OR: 7.79; CI: 6.88–8.70, *p* < 0.000). This finding remains stable also after controlling for several sociodemographic, clinical, contextual and gynecological variables. Moreover, when exploring the interaction time^*^presence of MB, this risk is almost four times higher 1 months after delivery (OR: 4.66; CI: 2.54–6.75, *p* < 0.000), almost three times higher after 3 months (OR: 2.98; CI: 0.50–5.46, *p* < 0.01) and almost six times higher after 12 months (OR: 5.88; CI: 3.20–8.54, *p* < 0.000). Other risk factors emerging from the linear regression model included presence of depressive disorders prior to pregnancy (OR:1.1; CI: −0.00–2.21, *p* < 0.05), conflicts with the partner (OR: 1.19; CI: 0.25–2.14; *p* < 0.01), having a partner with an anxiety disorder (OR: 1.09; CI: 0.20–1.99, *p* < 0.05), and increased fetal health rate during pregnancy (OR: 1.95; CI: −0.56–3.35, *p* < 0.01) (Table 3).

**Table 3:** Predictors of PDD at follow-ups (dependent variable: EPDS main score).

			**95% Confidence interval**	
	**OR**	***P*-value**	**Lower bound**	**Upper bound**
Intercept	14.6	0.000	10.44	18.795
**Time of assessment**				
One month	8.64	0.01	5.23	12.05
Three months	1.94	0.35	−2.18	6.06
Six months	0.26	0.99	−2.72	2.77
Twelve months	5.37	0.01	1.99	8.80
History of depressive disorder prior of pregnancy	1.10	0.05	0.00	2.21
History of anxiety disorders prior to pregnancy	−0.05	0.90	−0.88	0.77
Having a partner with an anxiety disorder	1.09	0.05	0.20	1.99
Tobacco smoking	−0.18	0.66	−0.98	0.61
Family conflicts	−0.27	0.56	−1.17	0.63
Conflicts with partner	1.19	0.01	0.25	2.14
Financial problems	−0.8	0.89	−1.25	1.09
Vaginal delivery	−0.39	0.10	−0.84	0.07
Preeclampsia	−0.54	0.33	−1.64	0.55
Increased fetal heart rate	1.95	0.01	−0.56	3.35
Presence of Maternity Blues, yes	7.79	0.000	6.88	8.70
Age	−0.27	0.15	−0.63	0.01
Apgar after 1 min	0.75	0.70	−0.31	0.46
Apgar after 5 min	0.156	0.12	−0.71	0.39
Time*maternity blues				
One month	4.66	0.000	2.57	6.75
Three months	2.978	0.01	0.50	5.46
Six months	0.33	0.76	−1.81	2.48
Twelve months	5.88	0.000	3.20	8.54

## Discussion

In this study we assessed which factors influence the presence of depressive symptoms immediately after delivery and we tested the hypothesis that the presence of maternity blues can be considered an independent risk factor for developing port-partum depression. To the best of our knowledge, this is the first study on the impact of MB on the risk to develop depressive symptoms during the entire post-partum period (i.e., up to 12 months after delivery) in a sample of Italian women.

We found that maternity blues was present in about 23% of women. The rates of MB in previous studies greatly vary, ranging from 13.7 to 76% (14). This heterogeneity may be due to the assessment instruments used to explore MB. In fact, in many studies MB was assessed retrospectively, or with instruments not specifically developed to assess MB (16). Moreover, when the EPDS had been used, different cut-offs were considered, i.e., ≥10 indicating the presence of depressive symptoms, ≥13 indicating severe depressive symptoms (38), or ≥9, with the potential inclusion of possible false positive women.

Moreover, the prevalence rates of MB greatly vary across countries and across cultures. In fact, the rates of MB are generally higher in low- and middle-income countries compared to high-income countries (39), being higher in Africa and Asia than in Europe and US. A recent metanalysis of all studies assessing MB reported a pooled prevalence rate of MB of 76% in low-income countries, of 40.8% in middle-income countries and of 38.4% in high-income countries, and a pooled prevalence rate of 29.4% among all studies using the EPDS, which overlaps with the prevalence rate found in our present study. The slight observed differences can be due to the higher level of education reported in our sample.

Concerning our second research question (i.e., which are those characteristics more frequently associated with MB), we identified several risk factors from the regression model. We found that the presence of an anxiety disorder in the past is a predictive factor for the onset of MB. A positive history for psychiatric disorders has been reported in retrospective, cross-sectional, and prospective studies as increasing the likelihood of affective disorders during the puerperium (40). The majority of studies have focused on the presence of previous affective episodes as risk factors for MB, while the finding that the presence of anxiety disorders is a risk factors for MB is rather new, although the impact of anxiety disorders on the onset of PPD had already been reported (41). It may be that women with anxiety disorders may perceive as more stressful the changes in their lifestyle behaviors imposed by the motherhood and feel less ready to take over the new role of mother (42). It has to be noted that, when assessing EPDS subscales immediately after the delivery, the levels of anxiety symptoms are notably higher compared to the levels of affective symptoms. This finding, which is in line with available data, suggests the importance to use the EPDS in the immediate post-partum in order to detect the presence of anxiety symptoms (43). In fact, anxiety disorders in the immediate post-partum can interfere with the development of a good mother-child bonding and can be associated with a higher risk of not initiating or continuing lactation (44).

In our sample, the presence of anxiety disorders in the partner during pregnancy (OR = 4.39, *p* < 0.01) and of family conflicts (OR = 6.06, *p* < 0.001) increase women's risk to have MB. These results are in line with previous data, reporting that environmental factors, such as poor marital relationship and lack of support from family members, play an important role in the onset of depressive symptoms after childbirth (40, 45). Women with a partner suffering from an anxiety disorder or with conflicts with other family members could feel under supported and perceive a greater feeling of loneliness. Moreover, it is worth noting that the role of paternal mental health in the perinatal period is poorly investigated. It has been reported that women whose partners suffered from a depressive episode have a 4-fold higher risk of having worse depressive symptoms, but the role of anxiety disorders in fathers has not yet been explored (46). These results highlight the importance of family relationship in the development of MB and strongly support the need to provide adequate psychosocial family interventions during pregnancy and in the immediate post-partum. In particular, psychoeducational family intervention are effective in a variety of psychiatric illnesses, including major depression, bipolar disorders and schizophrenia (47–50) in reducing symptom severity and improving family communication and problem-solving skills.

Finally, we found that MB is predicted by the presence of preeclampsia [defined as blood pressure exceeding 140/90 mmHg, together with proteinuria or any other organ involvement, Auger et al. (51)] (OR = 6.64, *p* < 0.01) and of increased fetal heart rate (OR = 9.15, *p* < 0.01). Medical complications occurring during pregnancy and/or childbirth have been inconsistently linked with postpartum psychiatric disorders, including MB (52). In particular, some studies have showed an association between postpartum psychiatric episodes and pre-eclampsia in women with MB (53, 54), while other studies did not found this association (55). More recently, Auger et al. (56) found that preeclampsia is associated with a modest increase in the risk of hospitalization for depression up to three decades after pregnancy, suggesting a shared biologic pathway between depression and preeclampsia, which includes inflammation and oxidative stress (57, 58).

Finally, we found that women with MB have a 7-fold increased risk of developing perinatal depression up to 1 year after delivery (*p* < 0.0001). In particular, when assessing the interaction between the presence of MB and the risk to develop PPD at follow-ups, we found that the risk is particularly higher after 1 month (OR = 4.66, *p* < 0.0001), 3 months (OR = 2.98, *p* < 0.01), and 12 months (OR = 5.88, *p* < 0.0001). This finding confirms the role of MB as a risk factor for the development of PPD, 1 month (22) and 3 months (3) after delivery. In our study we demonstrated that the predicting role of MB on the onset of PDD is still valid on a longer period, and therefore symptoms of MB should be closely monitored by health professionals in order to avoid long-term complications on the mothers. Training courses should be made available to both mental health professionals and other health care providers involved in mother care (i.e., primary physicians, obstetricians and gynecologists) (59, 60). Moreover, women with MB should receive appropriate pharmacological and psychosocial interventions in order to minimize the risk to develop PPD (61–63). Women with MB should be followed-up for at least the whole duration of postpartum (i.e., up to 1 year from the delivery), but even longer if MB symptoms don't fade away (64, 65).

The other risk factors for the development of PPD in the 12 months after the delivery include conflicts with the partner, having a partner with anxiety disorders, a personal history of depressive disorders needing pharmacological treatment. These risk factors support the existence of a “risk profile” for PDD (19), characterized by having MB, marital problems and a personal vulnerability to depression. These at-risk women should be supported with *ad-hoc* interventions that could be offered also as a virtual treatment (66–69).

Our study has some limitations. First, the small sample size. Since MB occurs in a minority of women, large sample sizes are needed to perform more appropriate analyses with robust findings. However, our sample size will be increased as the study is still ongoing. Moreover, the history of a psychiatric diagnosis in women and their partner has been retrospectively investigated. Another limitation is the fact that only 106 out of 359 women compiled the EPDS after 12 months. However, the drop-out rate of our sample is similar to other longitudinal studies carried out with similar methodologies.

In conclusion, the main finding of the present study highlights the fact that the presence of depressive symptoms immediately after the childbirth can be considered one of the most significant risk factors for the development of depressive symptoms up to 20 months after delivery. This result is particularly relevant both for clinicians and researchers, considering that in the majority of cases women with maternity blues do not seek help for their depressive symptoms and that professionals are not adequately trained to detect and treat them. Moreover, considering that depressive symptoms during post-partum can have several consequences also on child affective and cognitive development, our data support the need to identify new strategies to adequately treat these conditions and reduce the long-term negative impact on the mothers as well as on their babies and family members.

## Data Availability Statement

The raw data supporting the conclusions of this article will be made available by the authors, without undue reservation.

## Ethics Statement

The studies involving human participants were reviewed and approved by Ethical Review Board of the University of Campania Luigi Vanvitelli (protocol number 98 of February 28, 2019). The patients/participants provided their written informed consent to participate in this study.

## Author Contributions

MLu, AF, MT, and GS contributed to conception and design of the study. VD, VG, FP, MC, and MR organized the database. GS, MD, and MLa performed the statistical analysis. MLu and GS wrote the first draft of the manuscript. All authors contributed to manuscript revision, read, and approved the submitted version.

## Conflict of Interest

The authors declare that the research was conducted in the absence of any commercial or financial relationships that could be construed as a potential conflict of interest.

## Publisher's Note

All claims expressed in this article are solely those of the authors and do not necessarily represent those of their affiliated organizations, or those of the publisher, the editors and the reviewers. Any product that may be evaluated in this article, or claim that may be made by its manufacturer, is not guaranteed or endorsed by the publisher.
